# Is combined use of intravenous and intraarticular tranexamic acid superior to intravenous or intraarticular tranexamic acid alone in total knee arthroplasty? A meta-analysis of randomized controlled trials

**DOI:** 10.1186/s13018-017-0559-2

**Published:** 2017-04-18

**Authors:** Bobin Mi, Guohui Liu, Huijuan Lv, Yi Liu, Kun Zha, Qipeng Wu, Jing Liu

**Affiliations:** 1 Department of Orthopedics, Union Hospital, Tongji Medical College, Huazhong University of Science and Technology, 1277, Jiefang Avenue, Wuhan, China; 2 Department of Rheumatology, Tangdu Hospital, The Fourth Military Medical University, 1, Xinsi Avenue, Xi’an, China

**Keywords:** Tranexamic acid, Combined, Intravenous, Intraarticular, Total knee arthroplasty

## Abstract

**Background:**

Tranexamic acid (TXA) has been proven to be effective in reducing blood loss and transfusion rate after total knee arthroplasty (TKA) without increasing the risk of deep vein thrombosis (DVT) and pulmonary embolism (PE). Recently, an increasing number of studies have been interested in applying combined intravenous (IV) with intraarticular (IA) tranexamic acid in total knee arthroplasty. The purpose of this meta-analysis was to compare the blood loss and complications of combined TXA with IV TXA or IA TXA on TKA.

**Methods:**

Systematic search of literatures were conducted to identify related articles that were published in PubMed, MEDLINE, Embase, the Cochrane Library, SpringerLink, ClinicalTrials.gov, and Ovid from their inception to September 2016. All studies that compare blood loss and complications of combined TXA and IV TXA or IA TXA on TKA were included. Main outcomes were collected and analyzed by the Review Manager 5.3.

**Results:**

Five studies were included in the present meta-analysis. There was significant difference in total blood loss and blood volume of drainage when compared combined TXA group with IV TXA group or IA TXA group (*P* < 0.05). There was no difference in transfusion rate and thromboembolic complications when comparing combined TXA with IV TXA or IA TXA alone (*P* > 0.05).

**Conclusions:**

Compared with administration of IA TXA or IV TXA alone on TKA, combined use of TXA has advantages in reducing total blood loss and blood volume of drainage without increasing the incidence of thromboembolic complications. We recommend combined TXA as the preferred option for patients undergoing TKA.

## Background

As the number of patients who were afflicted with osteoarthritis (OA) is steadily increasing, the surgical volume of primary total knee arthroplasty (TKA) is increasing as well [1]. However, primary TKA is closely associated with the increase of total blood loss and transfusion rate. Some studies reported that the total blood loss can reach to 1500 mL and 60% of patients need allogeneic blood transfusion [2, 3]. Massive blood transfusion requirements in TKA increased the risks of allergic reaction, immune response, cost, and infection [4, 5]. Various blood-conserving techniques have been used to reduce blood transfusion, including controlled hypertension, tourniquet, and tranexamic acid (TXA) [6–8].

It has been widely accepted that patients undergoing TKA have an increased risk of perioperative bleeding [9]. TXA is an antifibrinolytic drug that inhibits the activation of plasminogen so as to decrease the amount of blood loss [10]. TXA can be applied by the intravenous (IV) or the intraarticular (IA) route. However, to achieve the maximum plasma concentration, TXA takes about 5–15 min for IV administration and 30 min for IA administration. Thus, IV administration is a rapid route for patients to increase the therapeutic concentration of TXA. Then, an increasing number of studies began to pay close attention on the effect of IV TXA on TKA [11, 12]. It was reported that IV TXA decreased perioperative bleeding and caused a reduction in total blood loss by up to 32%. Compared with IV TXA, the IA TXA has some advantages, such as easy administration, providing a maximum concentration of TXA at the bleeding site and inhibiting local activation of fibrinolysis [13]. Recent studies have confirmed that the administration of TXA, which is used directly into the surgical wound, reduced postoperative bleeding from 20 to 25% [13].

Recently, more and more studies tended to use combined TXA instead of using IV or IA TXA alone [14, 15]. It was shown that this method (combined TXA) can effectively reduce the amount of bleeding after TKA. Nevertheless, these studies reported inconsistent results of comparing combined TXA with IV or IA TXA alone on TKA [15–17]. Therefore, this meta-analysis was designed to compare the effectiveness and safety of combined TXA with IV TXA or IA TXA for patients undergoing primary TKA through evaluating the total blood loss, blood volume of drainage, transfusion rate, and thromboembolic complications.

## Methods

### Search strategy

Articles were searched in the following databases from their inception to September 2016: PubMed, MEDLINE, Embase, the Cochrane Library, SpringerLink, ClinicalTrials.gov, and Ovid. The following search terms were used: tranexamic acid or TXA or topical tranexamic acid or topical TXA or intraarticular tranexamic acid or IA TXA or intravenous tranexamic acid or IV TXA or total knee arthroplasty or TKA or total knee replacement or TKR.

### Data selection

To evaluate eligibility for inclusion, two investigators screened the title and abstracts of the articles independently. Any disagreements were resolved by discussion among the authors. A third researcher was the adjudicator when there were debates between two investigators. Articles should meet the following criteria: (1) the studies should be designed as RCTs, (2) the participants should be at least 18 years old, (3) the articles should be comparing the combined TXA with IV or IA TXA, and (4) the articles were restricted to English language.

### Data extraction

Two authors independently extracted the following data from each eligible study: study design, type of study population, age, number of participants, and interventions. Discrepancies were resolved by a third investigator.

### Quality and risk of bias assessments

The modified Jadad scale was used to assess the methodological quality of each study. A score of ≥4 indicates high quality. The Cochrane Handbook for Reviews of Interventions (RevMan Version 5.3) was used to assess the risk of bias. Two authors subjectively reviewed all articles and assigned a value of “high,” “low,” or “unclear” based on the following: selection bias, performance bias, detection bias, attrition bias, reporting bias, and other bias. Any disagreements were resolved by discussion and consensus. In order to improve accuracy, a third investigator was consulted when any disagreement emerged.

### Statistical analysis

RevMan software was used to analyze the data from included studies. For binary data, risk ratio (RR) and 95% confidence interval (CI) were assessed ($\alpha$ = 0.05 for the inspection standards). For continuous data, means and standard deviations were pooled to a weighted mean difference (WMD) and 95% confidence internal (CI) in the meta-analysis. Heterogeneity was tested using the *I* ^2^ statistic. Studies with an *I* ^2^ statistic of 25 to 50% were considered to have low heterogeneity. Those with an *I* ^2^ statistic of 50 to 75% were considered to have moderate heterogeneity. Those with an *I* ^2^ statistic >75% were considered to have high heterogeneity. When the *I* ^2^ statistic was >50%, sensitivity analyses were performed to identify the potential sources of heterogeneity [18]. Statistical significance was indicated by a *P* value <0.05.

## Results

### Description of studies and demographic characteristics

**Fig. 1:**
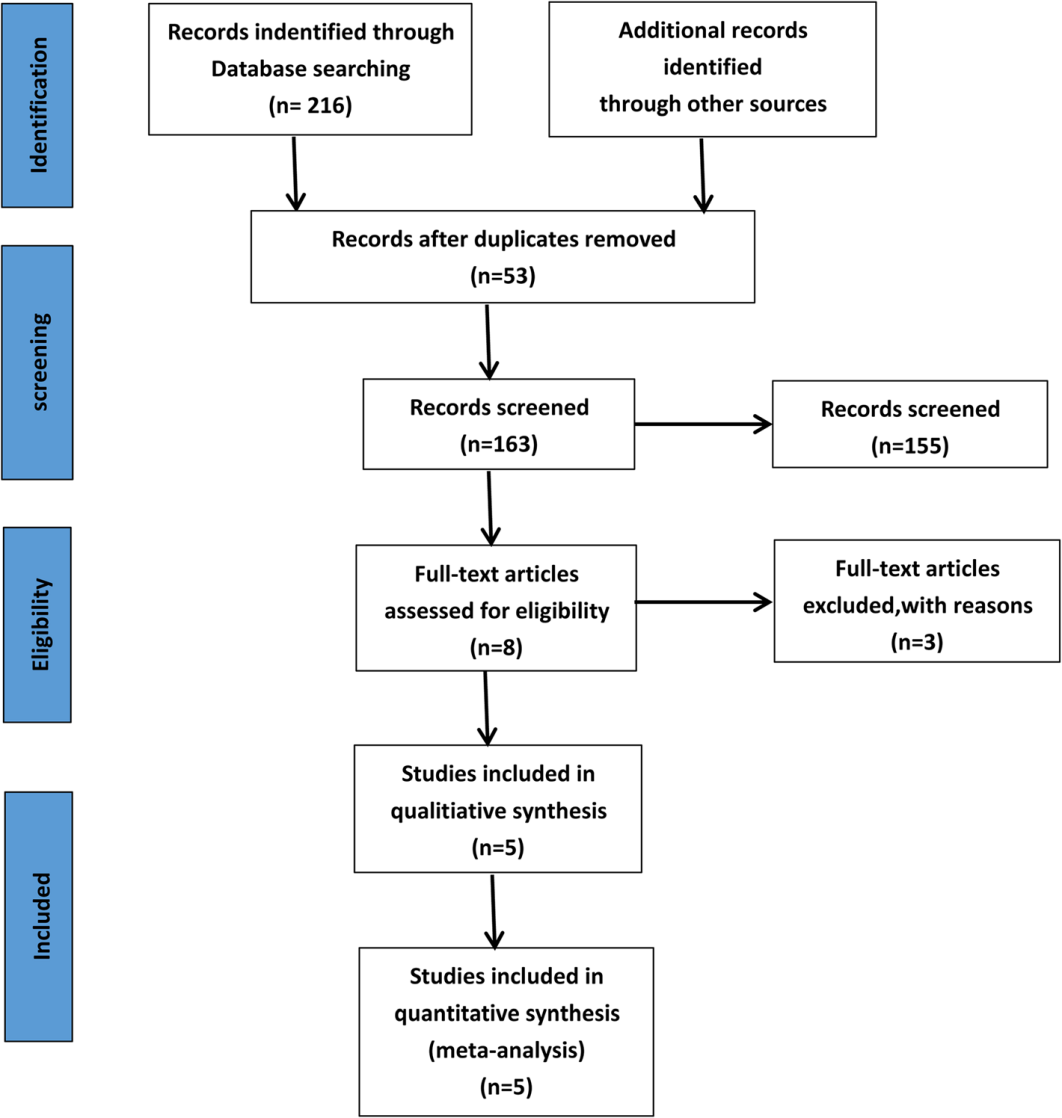
Flow chart showing study identification, inclusion, and exclusion

As shown in Fig. 1, a total of 216 articles were identified as potentially relevant studies. According to the agreed criteria, subsequent scrutiny leads to the exclusion of 208 citations. Full publications were obtained for eight citations: these were assessed and three further citations were excluded, leaving five trials included in the meta-analysis. The demographic characteristics were summarized in Tables 1 and 2. Among them, three trials [15, 17, 19] compared the effect of the combined TXA group with the IV TXA group, one trial [16] compared the combined TXA group with the IA TXA group, one trial [20] compared the combined TXA group with both the IA TXA group and the IA TXA group, respectively.

**Table 1:** The characteristics of included studies

Study	Year	Country	Patients (*n*)			Age (years)			Study design	Diagnosis	Quality score
			C	IA	IV	C	IA	IV			
Huang ZY	2014	China	92		92	65.4 ± 8.7		64.7 ± 9.5	RCT	OA	4
Jain NP	2016	India	59		60	68.2 ± 8.66		70.0 ± 6.56	RCT	OA	5
Lin SY	2015	Taiwan	40	40		70.7 ± 8.2	71.0 ± 7.2		RCT	OA	4
Nielsen CS	2016	Denmark	30		30	65.5 ± 7.8		63.2 ± 8.6	RCT	OA	7
Song EK	2016	Korea	50	50	50	70.8 ± 6.8	69.8 ± 6.8	69.2 ± 6.4	RCT	OA	7

**Table 2:** Characteristics of the five trials selected showing general intervention information

Study	Combine		IA	IV	Transfusion criteria	Pneumatic tourniquet	Thromboprophylaxis	DVT screening method
	IA	IV						
Huang ZY	1.5 g TXA 50 mL NS after implantation of the components	1.5 g TXA before inflation of the tourniquet		3 g TXA before inflation of the tourniquet	7.0 g/dL < HB < 10 g/dL + symptomatic anemia HB < 7.0 g/dL	Yes	LMWH	Doppler ultrasound
Jain NP	2 g TXA 30 mL NS 5 min before closure of arthrotomy	15 mg/kg TXA 30 min before skin incision 10 mg/kg TXA 3 and 6 h after surgery		15 mg/kg TXA 30 min before skin incision 10 mg/kg TXA 3 and 6 h after surgery	7.0 g/dL < HB < 8.0 g/dL + symptoms HB < 7.0 g/dL	No	Aspirin	Ultrasonographic + clinical symptom
Lin SY	1 g TXA after joint capsule closure	1 g TXA 15 min before skin incision	1 g TXA 20 mL NS after joint capsule closure		HB < 8.0 g/dL HB < 9.0 g/dL +symptoms	Yes	Rivaroxaban	Clinical symptom
Nielsen CS	3 g TXA 100 mL NS after closure of the capsule	1 g TXA preoperative		1 g of TXA	HB < 7.5 g/dL HB < 10 g/dL +symptoms postoperative Hb level was reduced >25% + symptoms	No	Rivaroxaban	NS
Song EK	1.5 g TXA 50 mL NS after wound close	10 mg/kg 20 min before tourniquet application 10 mg/kg 3 h after the second dose	1.5 g TXA 50 mL NS after wound closure	10 mg/kg 20 min before tourniquet application 10 mg/kg 15 min before deflation of the tourniquet 10 mg/kg 3 h after the second dose	HB < 8 g/dL	Yes	LMWH	Clinical symptom + Doppler ultrasonography and CT angiography

### Risk of bias in included studies

**Fig. 2:**
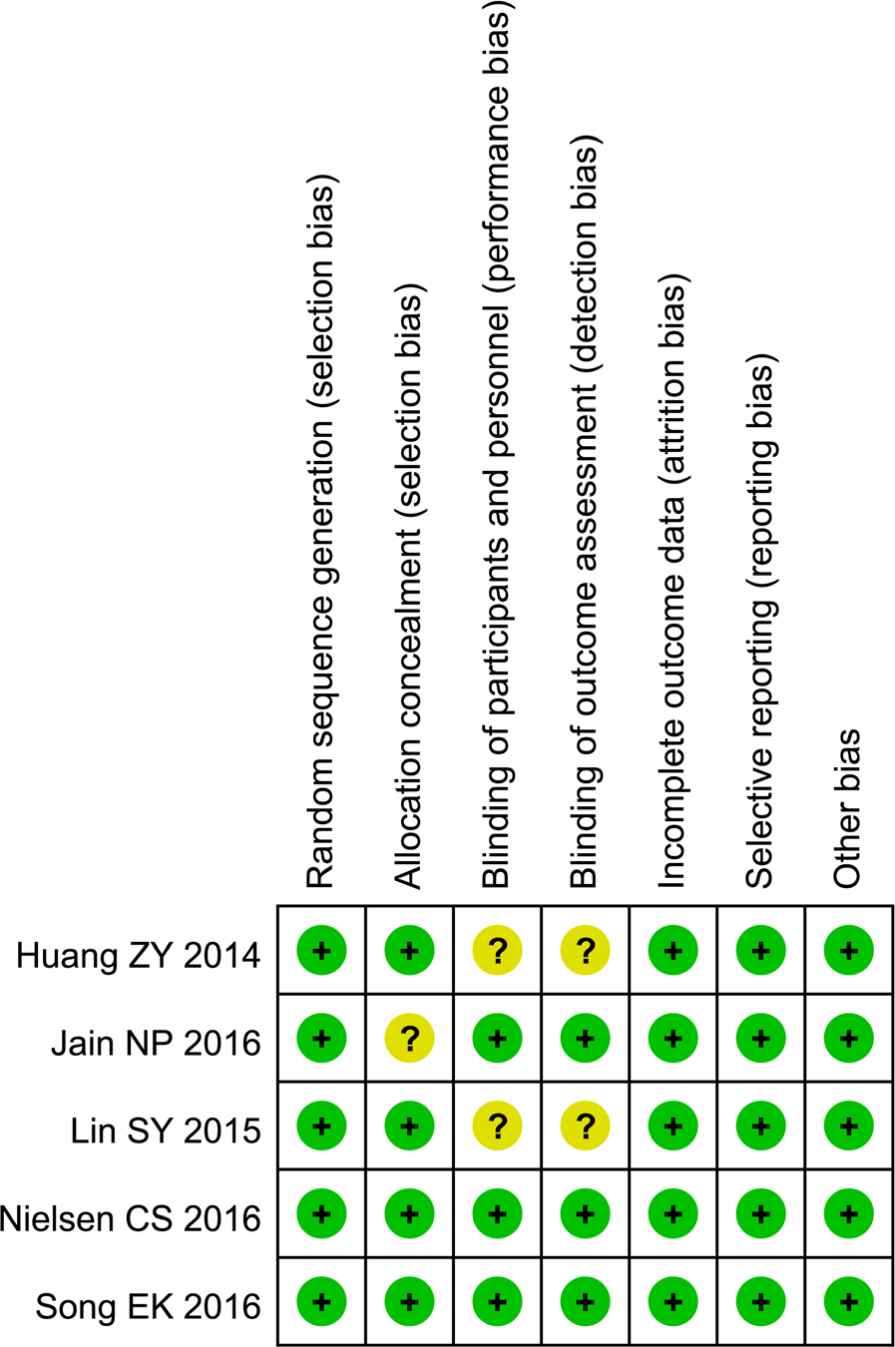
Risk of bias summary

The assessment of risk of bias was presented in the “Risk of bias assessment of included studies” (Fig. 2). All trials were described as randomized trial design. One trial [15] did not show detailed information of random sequence generation, and one trial [19] did not describe the methods of allocation concealment. Blinding of participants and personal (performance bias) were considered to be unclear in two trials [15, 16].

### Sensitivity and heterogeneity analysis

There was significant heterogeneity for the impact of IV TXA application on total blood loss. The leave-one-out analysis showed that the key contributor to this high heterogeneity was one study conducted by Jain et al. [19]. After excluding it, heterogeneity was reduced to *I* ^2^ = 73% for total blood loss. But significance of the pooled changes was not altered, which demonstrated that the results were robust.

### Total blood loss

**Fig. 3:**
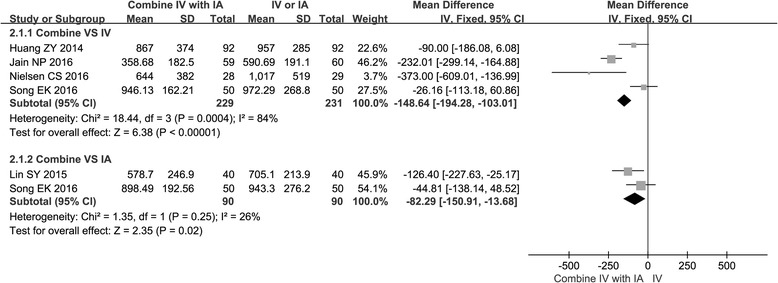
Forest plot of total blood loss when comparing the combined group with the IV group or the IA group

All of the studies provided the data of total blood loss. Three studies [15, 17, 19] reported data of the combined group compared with the IV group, one study [16] reported data of the combined group compared with the IA group, and one study [20] reported data of the combined group compared with both the IV group and the IA group respectively. There was significant difference in terms of reducing total blood loss when comparing the combined group with the IV group (chi^2^ = 18.44, *I* ^2^ = 84%, *P* < 0.05) or the IA group (chi^2^ = 1.35, *I* ^2^ = 26%, *P* < 0.05) (Fig. 3).

### Blood volume of drainage

**Fig. 4:**
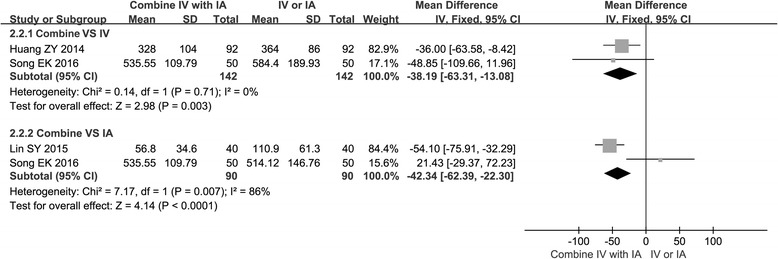
Forest plot of blood volume of drainage when comparing the combined group with the IV group or the IA group

Three studies [15, 16, 20] provided data on blood volume of drainage. The result of the blood volume of drainage illustrated significant difference in the combined group when compared with the IV group (MD −38.19, 95% CI −63.31 to −13.08, *P* < 0.05, *I* ^2^ = 0%). There was significant difference in blood volume of drainage between the combined group and the IA group (MD −42.34, 95% CI −62.39 to −22.30, *P* < 0.05); however, this result should be interpreted with caution due to the presence of statistical heterogeneity (chi^2^ = 7.17, *I* ^2^ = 86%) (Fig. 4).

### Transfusion rate

**Fig. 5:**
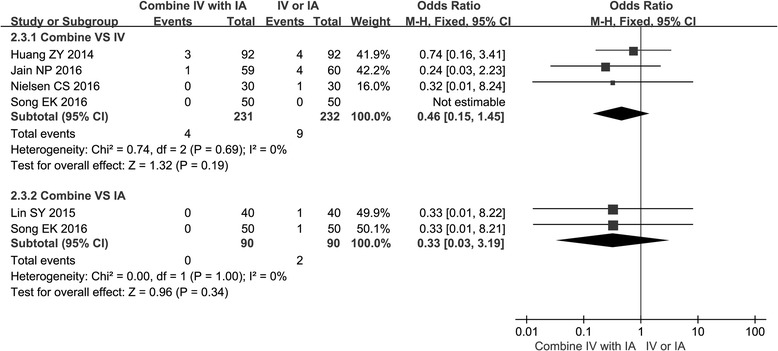
Forest plot of transfusion rate when comparing the combined group with the IV group or the IA group

The data of transfusion rate was provided from all of the studies. There was no significant difference in transfusion rate when comparing the combined group with the IV or the IA group (chi^2^ = 0.74, *I* ^2^ = 0%, *P* > 0.05; chi^2^ = 0, *I* ^2^ = 0%, *P* > 0.05) (Fig. 5).

### Thromboembolic complications

**Fig. 6:**
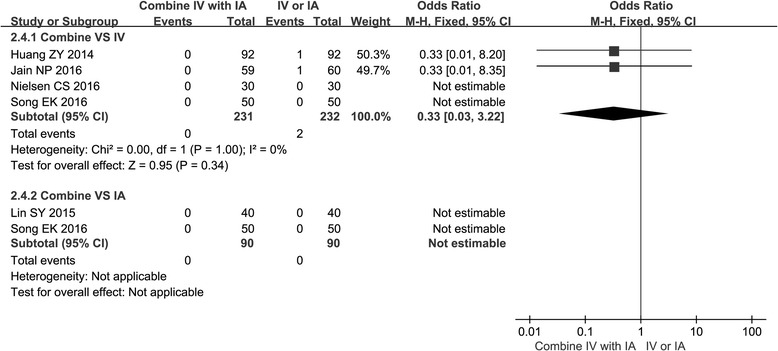
Forest plot of thromboembolic complications when comparing the combined group with the IV group or the IA group

All of the studies provided data of thromboembolic complications. There was no significant difference in thromboembolic complications when comparing the combined group with the IV or the IA group. There was only one study that reported one case of DVT in the IV group [15]. Song [20] reported that there were three patients from the IV group and two patients from the combined group and one patient from the IA group had clinical suspicion of DVT based on calf swelling and tenderness. One case of clinical suspicion of DVT in the IV group was reported by Jain et al. [19] (Fig. 6).

## Discussion

TXA is an antifibrinolytic agent which has been widely used today. Several studies have reported that IV administration of TXA can effectively reduce total blood on TKA [21]. Compared with IV TXA, the IA administration of TXA has the advantage of reaching to a maximum concentration of TXA at the bleeding site, and it is associated with low systemic absorption [22]. Considering the advantages of both IV TXA and IA TXA, it is logical to suggest that combined use of IV TXA and IA TXA is a more efficient method of reducing total blood loss. Karaaslan et al. [23] reported that combined use of TXA in patients undergoing TKA can reduce blood loss with negligible side effects. Other studies [15, 16] that compared combined TXA with IV or IA TXA also reported that combined TXA was more effective than IV or IA TXA alone for patients undergoing TKA. In this meta-analysis, when compared with the IV group, we found that the combined group had reduced total blood loss by a mean of −148.64 mL (CI −194.28 to −103.01), and when compared with the IA group, it reduced total blood loss by a mean of −82.29 mL (CI −150.91 to −13.68). These results confirmed that combined TXA was more efficient for patients undergoing TKA in terms of reducing total blood loss.

Previous studies and several meta-analysis of IV TXA showed that administration of TXA intravenously reduced blood volume of drainage by up to 50% [12, 24]. Since IA administration of TXA can inhibit local activation of fibrinolysis and reduce time to vascular occlusion [25], it also has the advantage of limiting local blood loss [26]. Then, combined use of TXA has the advantages of both IV TXA and IA TXA in terms of reducing blood volume of drainage. Our meta-analysis also suggested that TXA administration that used the combination method resulted in a lower blood volume of drainage than TXA administration that used IV or IA alone (−38.19 and −42.34 mL, respectively, *P* < 0.05). Interestingly, there was a slightly higher transfusion rate in the combined group when compared with the IV or the IA group (0.46, 0.33, respectively), even though there was no significant difference (*P* > 0.05). These results may be attributed to the fact that the transfusion criteria were inconsistent among these studies. In addition, a limited number of RCTs and patients may also lead to these results.

It is well known that patients undergoing TKA will take risks of DVT or PE [27, 28]. TXA has been widely used in TKA, while the risk of thromboembolic events are increasingly concerned [29]. Our meta-analysis had shown that there was no significant difference in thromboembolic complications when comparing the combined group with the IV or the IA group. This result was consistent with those studies that recommend the use of combined TXA on TXA [15, 19]. One highly observable time of DVT or PE was the postoperative of TKA within 30 days [30], and chemoprophylaxis [31, 32] has been recommended to those patients. All of our studies observed the DVT or PE at least 30 days, and chemical prophylaxis was given to all patients or to those high-risk patients. Only one case of DVT was detected in the IV group 3 days after operation [15]. It should be noted that pneumatic tourniquet application could increase the risk of DVT or PE [33]. All patients reported by Huang et al. [15] used pneumatic tourniquet, and one case of DVT was detected in the IV group. In addition, Song [20] reported that three patients from the IV group, two from the combined group, and one patient from the IA group had clinical suspicion of DVT based on calf swelling and tenderness. Then, which one was the main reason for increased DVT or PE, TXA, or pneumatic tourniquet? The reason should be further confirmed. In addition, some included studies [19, 20] had evaluated symptomatic patients only, which may have caused a lower incidence of thromboembolic complications and missed the real patients who have DVT or PE. Considering the above factors, the results need to be further confirmed.

There was significant heterogeneity in the administration of IV TXA on total blood loss. The leave-one-out analysis showed that the key contributor to this high heterogeneity was one study conducted by Jain et al. [19]. After excluding this study, heterogeneity was reduced to *I* ^2^ = 73% for total blood loss. By comparing these four studies that compared the combined group with the IV group, we found that the total blood loss in this study was calculated by hemoglobin balance method, whereas the total blood loss in other studies was calculated by gross formula [34]. Therefore, we infer that the calculation formula of total blood loss might be partly responsible for the heterogeneity.

There are several limitations in this meta-analysis. Firstly, the present meta-analysis focused only on papers published in English; the ones that were reported in other languages may increase heterogeneity and change the present results. Secondly, because four studies included subjects coming from Asia and one from Europe, the results cannot be extended to populations elsewhere. Besides, the dose and the timing of administrate IV TXA or IA TXA in the combined group were inconsistent among those studies. Further rigorously designed RCTs with larger sample sizes are needed to confirm the efficacy of combined TXA in primary TKA.

## Conclusions

Present meta-analysis results demonstrated that combined use of TXA in TKA significantly reduce the total blood loss and blood volume of drainage without increasing the adverse effect of DVT or PE. Further studies are needed to investigate an appropriate dose and times of administering IV TXA combined with IA TXA in patients undergoing TKA.

## Abbreviations

IA: Intraarticular
IV: Intravenous
OA: Osteoarthritis
TKA: Total knee arthroplasty
TXA: Tranexamic acid
